# ZoomHead: A Flexible and Lightweight Detection Head Structure Design for Slender Cracks

**DOI:** 10.3390/s25133990

**Published:** 2025-06-26

**Authors:** Hua Li, Fan Yang, Junzhou Huo, Qiang Gao, Shusen Deng, Chang Guo

**Affiliations:** School of Mechanical Engineering, Dalian University of Technology, Dalian 116024, China; lihua0107@mail.dlut.edu.cn (H.L.); gao13qiang88@163.com (Q.G.); dss22304037@mail.dlut.edu.cn (S.D.); guochang@mail.dlut.edu.cn (C.G.)

**Keywords:** metal surface crack, lightweight detection head, GroupNorm2d, DEConv, shared convolution structure

## Abstract

Detecting metal surface crack defects is of great significance for the safe operation of industrial equipment. However, most existing mainstream deep-object detection models suffer from complex structures, large parameter sizes, and high training costs, which hinder their deployment and application in frontline construction sites. Therefore, this paper optimizes the existing YOLO series head structure and proposes a lightweight detection head structure, ZoomHead, with lower computational complexity and stronger detection performance. First, the GroupNorm2d module replaces the BatchNorm2d module to stabilize the model’s feature distribution and accelerate the training speed. Second, Detail Enhanced Convolution (DEConv) replaces traditional convolution kernels, and shared convolution is adopted to reduce redundant structures, which enhances the ability to capture details and improves the detection performance for small objects. Next, the Zoom scale factor is introduced to achieve proportional scaling of the convolution kernels in the regression branch, minimizing redundant computational complexity. Finally, using the YOLOv10 and YOLO11 series models as baseline models, ZoomHead was used to replace the head structure of the baseline models entirely, and a series of performance comparison experiments were conducted on the rail surface crack dataset and NEU surface defect database. The results showed that the integration of ZoomHead effectively improved the model’s detection accuracy, reduced the number of parameters and computations, and increased the FPS, achieving a good balance between detection accuracy and speed. In the comparative experiment of the SOTA model, the addition of ZoomHead resulted in the model having the smallest number of parameters and the highest FPS, while maintaining the same mAP value as the SOTA model, indicating that the ZoomHead structure proposed in this paper has better comprehensive detection performance.

## 1. Introduction

Crack defect detection is crucial for ensuring safe operation and improving the performance of engineering structures. The crack morphology of structures subjected to complex loads is complex and diverse, developing rapidly and causing significant harm, which is difficult to detect and is an important hidden danger leading to structural damage and even catastrophic accidents. Scholars have extensively researched crack defect detection to prevent sudden structural failure and extend service life. Wu et al. [1] proposed an enhanced fluorescent magnetic particle inspection method to address the issue of obstructed crack training data. Lei et al. [2] considered the hidden danger of cracks on the inner wall of turbine blades in aircraft engines and designed a high-sensitivity eddy current probe for detecting cracks on the inner walls of blades. Yang et al. [3] proposed a new flexible ultrasonic, infrared, non-destructive testing method to meet the detection needs of complex geometric metal surface crack defects that can accurately detect cracks of different lengths. Ma et al. [4] designed an image acquisition module based on machine vision methods to solve the problem of online detection of surface cracks in stamped parts. Guclu et al. [5] proposed a defect segmentation model based on deep learning to improve surface crack defect detection accuracy and efficiency in steel structures. Hou et al. [6] proposed a crack detection model for aircraft engine blades based on an improved YOLOv5s structure, which reduces the number of parameters by 52% and significantly improves accuracy and convergence speed. Zheng et al. [7] proposed an attribute-weighted naive Bayes-improved OTSU algorithm to address the challenge of detecting cracks on metal surfaces under high overload impact. Image processing can quantitatively characterize cracks and achieve efficient detection. The above-mentioned scholars have researched crack defect detection from different perspectives using various methods. Among them, the high-precision and widely applicable image vision crack detection method has been increasingly valued by scholars with the development of deep learning.

Object detection can achieve precise localization of target boundaries in a given image and detect the category to which the target belongs. It is a research hotspot in the field of computer vision and is commonly used in defect detection [8], road detection [9], robot vision [10], and other fields. It is mainly divided into traditional object detection and deep learning-based object detection algorithms. The object detection algorithm based on deep learning relies on the powerful feature extraction ability of convolutional neural networks, which directly learn the features of the original image and efficiently complete the detection task. It can be divided into two-stage detection and single-stage detection. The former first generates candidate regions, and then classifies and regresses the candidate regions. Representative algorithms include R-CNN [11] and Faster R-CNN [12]. Single-stage detection directly predicts the target category and position, reducing task complexity and increasing efficiency. Representative algorithms include SSD [13] and YOLO [14]. Among them, the YOLO algorithm treats the target task as a unified, end-to-end regression problem and processes the image only once to determine the classification and position of the target. Its advantages of fast detection speed and high accuracy make it widely used in defect detection in industrial production, especially for metal components. Su et al. [15] proposed a cascaded combination method to solve the problem of detecting defects on metal gear end faces. By using 16X to extract regions from sampled features and multi-scale fusion, the method automatically detects defects with a recall rate of up to 97%. To improve the detection accuracy of steel surface defects, Li et al. [16] embedded an attention mechanism in the YOLOv4 backbone network to enhance the network’s feature extraction capability, resulting in a 3.87% increase in the average accuracy of the model. Wu et al. [17] integrated a multi-scale feature fusion attention module and a lightweight CSP structure into the YOLOX algorithm to solve the problem of the high missed detection rate of surface defects in steel. Hu et al. [18] proposed a faster and more effective defect-detection model for lithium battery steel shells, Sim-YOLOv5s, based on YOLOv5, which improved the detection accuracy by 6.9%. The research on metal surface crack defect detection based on the YOLO algorithm mentioned above shows a certain degree of improvement in detection accuracy compared with traditional detection methods. However, the requirement for high-precision detection makes the structure of the target detection algorithm more complex, and the parameters gradually reach a certain scale. The network model calculation increases, the real-time detection speed is slow, and higher requirements are also put forward for the operating equipment, which affects the feasibility of algorithm equipment deployment.

Given the problems of slow real-time reasoning of model crack detection, the high computational power dependence of model-training equipment, and the complex deployment of edge computing equipment models in metal surface crack defect detection, scholars have carried out research on lightweight algorithm structures and proposed a new algorithm combining the YOLO model and lightweight network to improve the feasibility of model deployment and operation in low-computational-power edge computing equipment. Zhang et al. [19] replaced the YOLOv5s backbone network with the MobileNetv3 lightweight network to balance the model’s light weight and accuracy and improve its fusion speed and efficiency. Yan et al. [20] made lightweight improvements to YOLOv5 by using ShuffleNetV2, which has higher computational efficiency, as the backbone network. The model parameter count was reduced by 44%, improving the efficiency and practicality of metal gear surface defect detection. Ma et al. [21] built YOLO-DCSAM based on YOLOv4 for detecting surface defects on aluminum strips, redesigned and lightweighted the neck network, and increased the detection speed by three times. Ni et al. [22] proposed a metal surface defect detection algorithm, FMR-YOLO, based on the improved YOLOv8n, which adopts a lightweight structure to reduce the number of model parameters and designs a receptive field attention structure in the neck section to reduce computational complexity. The above-mentioned research on lightweight object detection mainly focuses on the optimization design of the backbone network structure and the neck network structure. These improvements have improved the model’s detection speed and inference efficiency and reduced the demand for computing resources. However, an in-depth analysis of the computational distribution of typical deep learning object-detection models (YOLOv5n, YOLOv8n, YOLOv10n, YOLOv11n) reveals that the detection head dominates the overall computational complexity of the model. As shown in Table 1, the computational contribution of the head structure in multiple models exceeds 40% (some models even exceed 55%). Existing research on lightweight design and optimization of the detection head structure is relatively scarce, and most works still use traditional dense convolutional detection head designs, which have significant computational redundancy in feature decoding and bounding box regression processes. Therefore, targeted design and lightweight detection head structure improvement are of great value and significance for reducing model computation, improving detection performance, and deploying models on edge detection devices.

In summary, this paper proposes the YOLO-ZoomHead algorithm for detecting small and narrow metal cracks based on the YOLO algorithm. The contributions of this paper are as follows:

(1) Based on the existing YOLO series head structure, an innovative fusion of shared convolution and heavy parameterization technology is used to construct the YOLO-ZoomHead lightweight detection head. (2) To improve the normalization module, introducing the GroupNorm2d module instead of the BatchNorm2d module solves the problem of unstable BatchNorm2d performance, effectively improves the stability of feature distribution during model training and inference, accelerates training convergence speed, and enhances model generalization ability. (3) To optimize the convolutional structure, the traditional Conv convolution kernels are replaced with DEConv, incorporating the idea of shared convolution to reduce redundant structures and improve detection performance. At the same time, the Zoom scaling factor is introduced to perform relatively free proportional scaling operations on the convolution kernels in the regression branch, reducing redundant computational complexity and effectively reducing computational costs while ensuring detection accuracy. (4) A series of performance comparison experiments were conducted by deploying ZoomHead in baseline models such as YOLOv10 and YOLO11 (replacing the original detection head structure). The experimental results showed that compared with the baseline model, the addition of ZoomHead significantly reduced the computational complexity of the detection head and effectively improved the mAP value. (5) In the comparative experiment with the current SOTA model, the YOLO-ZoomHead model demonstrated the best overall performance. This model not only has the smallest number of parameters and the highest FPS, but also has mAP values that are on par with the SOTA model, fully demonstrating that YOLO-ZoomHead can effectively improve the detection performance of the model.

## 2. Related Works

### 2.1. Head Structure Function

**Figure 1:**
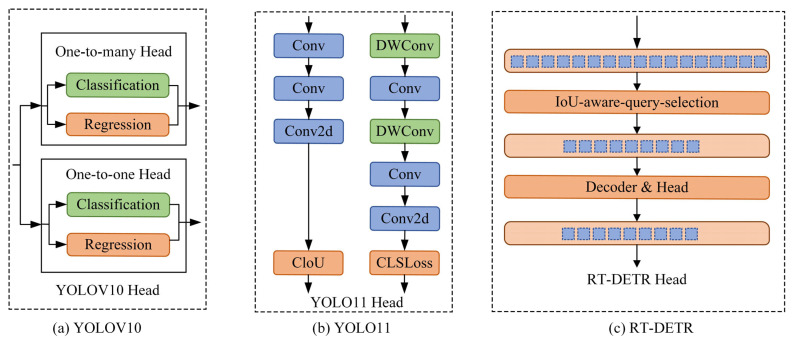
Head structure diagram.

As one of the key modules of the object detection model, the head structure is responsible for extracting features from the backbone and neck, decoding and converting them into spatial position and category confidence of the target, and performing multi-scale feature fusion to adapt to the detection of targets of different sizes. It can transform abstract features into concrete detection outputs, directly affecting the model’s detection performance and inference efficiency. Due to its design flexibility, computational efficiency, and end-to-end optimization advantages, it has become an innovative point for algorithm iterative improvement. The head structures of YOLOv10, YOLO11, and RT-DETR are shown in Figure 1. The YOLOv10 [23] model was proposed by Tsinghua University, which introduces a dual label allocation strategy, namely one-to-many label and one-to-one label allocation strategies. The head structure of YOLOv10 combines a one-to-many head and a one-to-one head lightweight detection head for training, and only a one-to-one head is needed for inference and prediction, eliminating non-maximum suppression and improving the inference speed and detection efficiency of the model. The YOLO11 [24] model was released by Ultralytics, which optimized the width and depth of the model based on YOLOv8. The head structure used YOLOv8′s decoupling head, and two depthwise separable convolutions were added to the classification detection head to ensure detection accuracy while significantly reducing computational complexity. The RT-DETR [25] model proposes a detection method that balances efficiency and real-time performance to address issues such as difficulty training DETR and slow convergence. The head network adopts a Transformer decoder architecture with integrated auxiliary prediction heads, combined with IoU-aware query selection technology to filter a fixed number of image features from the feature sequence output by the encoder as the initial object query of the decoder, which is input into the Transformer decoder. The prediction head queries and iteratively optimizes the target, generates categories and bounding boxes, and achieves real-time object detection.

### 2.2. Exploration and Improvement Work Around the Head Structure

To further improve the detection performance of object detection algorithms and reduce computational complexity, scholars have begun to explore lightweight design and improvement work for head structures. Huang et al. [26] improved the detection head module of YOLO to improve the speed of steel surface defect detection. They used convolutional compression and excitation modules to construct the SSA-YOLO model, significantly improving detection efficiency and accuracy. Wang et al. [27] proposed a steel defect detection algorithm based on YOLOv8, designed a RexSE head detection head structure, and weighted channel information to reduce computational redundancy, enhancing the sensitivity of crack defect detection. Liang et al. [28] proposed an improved metal crack defect detection model based on YOLOv5, which uses lightweight convolution combined with the VOV-GSCSP module to optimize the detection head structure of YOLOv5, reducing model parameters by 43.2%. Chen et al. [29] improved YOLOv8 from three key aspects—head, neck, and data—proposing an adaptive structural head to alleviate feature space misalignment between classification and regression tasks and improve detection accuracy, inference speed, and generalization performance. Zhao et al. [30] proposed an improved lightweight high-precision model YOLOv8-E, introducing DEConv technology into the head network to form a lightweight shared convolution object detection head module. By integrating prior information and adopting a shared convolution strategy, the model’s generalization ability is enhanced, and the model parameters are reduced by more than 50%. In the research on lightweighting the metal crack defect detection head structure based on the YOLO algorithm mentioned above, scholars have made improvements in module fusion, structural design, and task optimization, effectively reducing model computation and improving detection performance.

The above-mentioned lightweight research work on the head structure has achieved good results, but there are still some problems. While the lightweight design compresses the computational load, it weakens the ability to capture small crack features, which can lead to an increase in the missed detection rate under complex working conditions. In response to the problem of difficulty in further improving the retention and utilization of crack feature information in detecting small and narrow metal crack defects, this study innovatively constructs a lightweight detection head structure, YOLO-ZoomHead, that combines shared convolution and heavy parameterization ideas. It can effectively improve the performance of detecting minor crack defects while reducing computational complexity.

## 3. Methods

As the core output structure of the entire model, the head structure needs to process feature map information from different scales of backbone and neck structures and classify and locate targets of different scales in the image. It is precisely because all feature map information will undergo a series of integration, computation, and processing in the head structure that it usually contributes nearly half of the overall computational workload of the model. Therefore, it is of great significance to further improve the training and inference speed of the model, explore the balance between model detection performance and computational cost, and explore a streamlined optimization method for the head structure.

To effectively reduce the computational resource requirements of current mainstream object detection models and achieve high-precision and low-latency completion of crack detection tasks in industrial construction environments based on embedded edge computing devices deployment, this paper will optimize the head structure in the YOLO architecture as an example to improve model detection performance while effectively compressing the computational load of the head structure and enhancing model response speed.

### 3.1. Replace BN with GN

**Figure 2:**
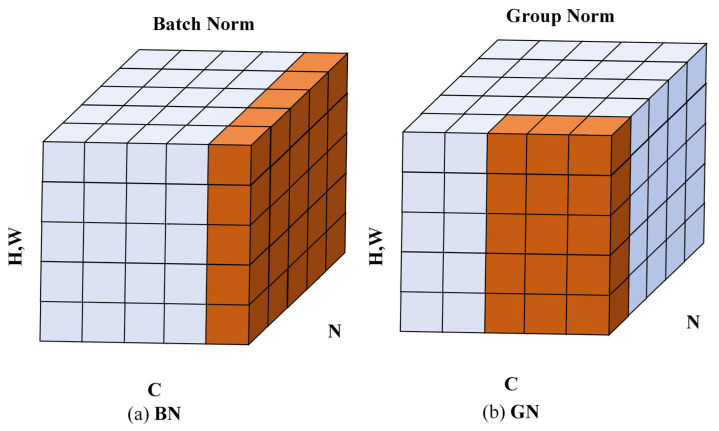
Schematic diagram of BN and GN three-dimensional features.

Adding normalization methods can achieve stable feature distribution, accelerate training convergence, and enhance model generalization ability in the training and inference process of mainstream object detection models. Batch Normalization (BN) is a common normalization method that solves the problem of input distribution changes in the middle layer of a network by normalizing the features within the batch, alleviating problems such as gradient vanishing and exploding and reducing the risk of overfitting. However, it relies too much on batch size, and when the training process takes small batch values, performance decreases due to insufficient normalized feature quantities. Group Normalization (GN) is another normalization method that significantly weakens the impact of batch size on training performance. GN groups channels and normalizes them within each group, which not only preserves some channel correlations but also has a more significant enhancement effect on detail features in object detection (such as edge features, small-scale targets, etc., which are more likely to be distributed within the same group). The mathematical expressions of BN and GN can be represented by Formulas (1)–(5). The three-dimensional feature maps of BN and GN are shown in Figure 2, where C represents the number of channels, N represents the batch size, and H and W represent the height and width, respectively.

To maximize model detection performance and reduce the additional impact of different batches on training effectiveness, this paper uses GN to replace the BN structure in the original head structure.

(1) $$\mu_{i}=\frac{1}{m}\sum_{k\in S_{i}}x_{k},$$

(2) $$\sigma_{i}^{2}=\frac{1}{m}\sum_{k\in S_{i}}{\left(x_{k}-\mu_{i}\right)}^{2},$$

(3) $$\tilde{x_{i}}=\frac{x_{i}-\mu_{i}}{\sqrt{\sigma_{i}^{2}+\varepsilon }}\cdot \gamma +\beta ,$$

In the formula: $\mu_{i}$ is the mean, $\sigma_{i}^{2}$ is the variance, γ and β are learnable scaling and offset parameters, respectively, and $S_{i}$ is the calculation area for mean and variance. In BN, it is

(4) $$S_{i}=\left\{k|k_{c}=i_{c}\right\}.$$

In GN, it is

(5) $$S_{i}=\left\{k|k_{N}=i_{N},floor\left(\frac{k_{C}}{C/G}\right),floor\left(\frac{i_{C}}{C/G}\right)\right\}.$$

In the formula, *C* represents the number of channels, and *N* is the batch size.

### 3.2. Detail-Enhanced Convolution

The traditional Conv convolution module is widely used in deep-object detection models to achieve spatial feature extraction, channel dimension adjustment, and receptive field control of targets in input images. However, traditional Conv also has some performance limitations. Its relatively inflexible receptive field makes it challenging to cover multi-scale targets or process complex contextual information uniformly. At the same time, due to the fixed parameters and single shape of the convolution kernel, its perceptual ability is limited when facing targets with complex geometric shapes.

**Figure 3:**
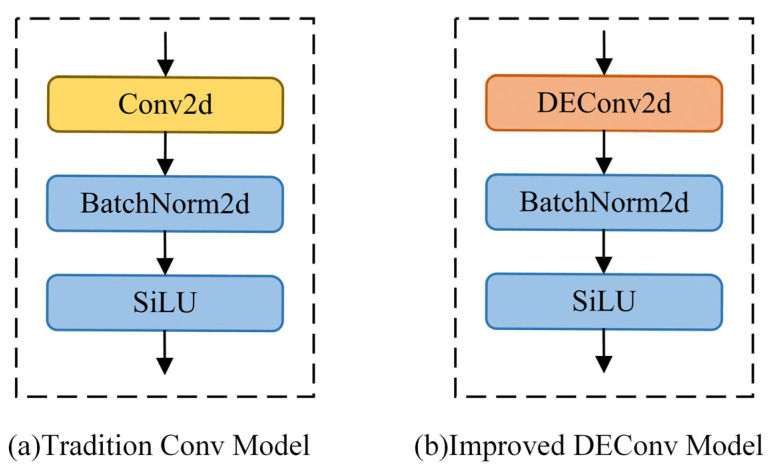
Schematic diagram of traditional Conv and improved DEConv structures.

Inspired by the idea of differential convolution, Chen et al. [31] proposed the DEConv module, which deploys four different directional differential convolutions (Center Differential Convolution (CDC), Angle Differential Convolution (ADC), Horizontal Differential Convolution (HDC), and Vertical Differential Convolution (VDC)) in parallel based on traditional Conv modules. By fully exploring and fusing local feature descriptions, DEConv can simultaneously capture multiple features of images, ultimately effectively enhancing CNN’s feature expression performance and model generalization ability. At the same time, to balance the complexity and feature extraction performance within the module, the DEConv module uses clever reparameterization techniques to optimize and adjust the network architecture, simplifying the five parallel but differently oriented convolution kernels within the module into a single ordinary convolution layer. Compared with traditional single Conv modules, DEConv modules not only do not generate additional parameters and computational complexity but also do not achieve performance improvement at cost. This article introduces the DEConv convolution kernel and replaces the traditional Conv convolution kernel as a whole. The improved structure is shown in Figure 3.

### 3.3. Overview of Head Structure

This section introduces the overall optimization scheme based on the YOLO model head structure. As mentioned earlier, the purpose of structural optimization is to reduce redundant structures and lower computational complexity.

**Figure 4:**
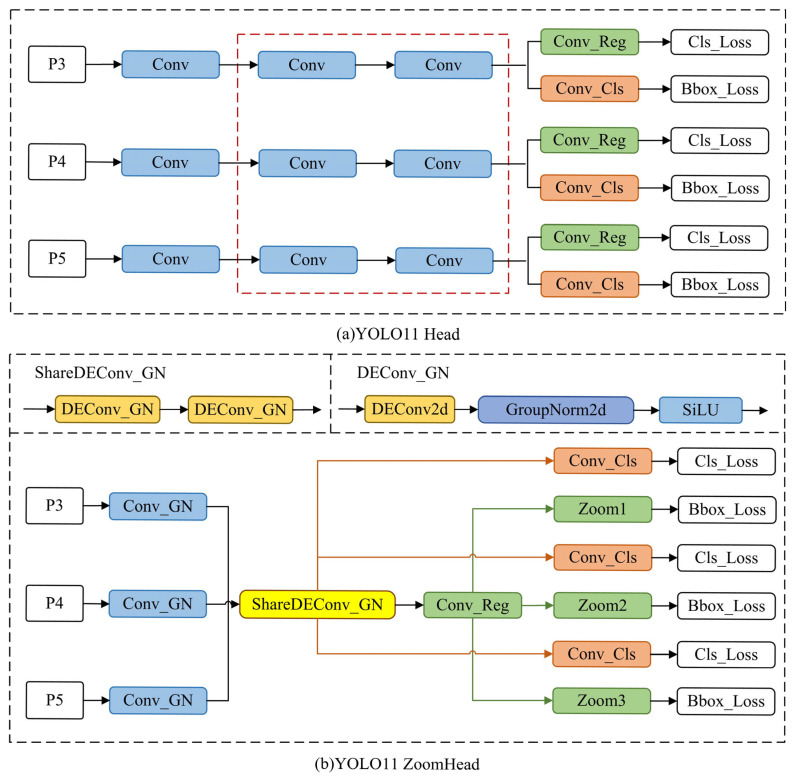
Schematic diagram of YOLO11 head and ZoomHead structure.

In the YOLO model head structure, multiple Conv modules are used for channel integration and the feature extraction of feature maps. The GN normalization method mentioned in 3.1replaces the BN normalization method of all Conv modules as a whole, denoted as Conv_GN. Since the head structure needs to process multiple feature map information of different scales (P3, P4, P5) in sequence, the three processing paths are independent, as shown in Figure 4a. After analysis, it was found that there is redundancy in the Conv module, marked by the red dashed box. Therefore, the idea of shared convolution kernels was introduced, and only one set of convolution kernels, using DEConv instead of traditional B, was set as the shared convolution kernel, denoted as ShareDEConv_GN. As shown in Figure 4b, the feature map information from (P3, P4, P5) passed through this set of convolution kernels in sequence, achieving the predetermined function and significantly reducing the redundant structure and computational load.

In addition, the head structure has regression branches for three different scale feature maps, as shown in Figure 4a. After analyzing the source code, it was found that this structure also has redundant calculations. Therefore, as shown in Figure 4b, the Zoom scale factor was introduced to provide relatively free proportional scaling operations for the convolution kernels in the regression branches. The obvious advantage is that only one set of convolution kernels needs to be set up and sequentially multiplied with three different Zoom values to achieve regression processing of information from three different scale feature maps, effectively reducing redundant computational complexity.

Based on the above two improvements, Figure 4b is a schematic diagram of the complete improved head structure proposed in this paper. The calculation process of the entire improved head structure can be represented by Equations (6)–(12). The specific content is that Equation (6) represents convolutional feature extraction, where $x_{i}$ is the *i*-th feature map, Conv_GN represents the convolution operation with GroupNorm2d normalization, and $x_{i}^{conv}$ represents the output feature map. Equation (7) represents the idea of using shared convolution kernels to extract features from $x_{i}^{conv}$ twice in a row using DEConv_GN. DEConv_GN represents the DEConv convolution operation with GroupNorm2d normalization, used for feature sampling or dimension transformation, and $x_{i}^{share}$ represents the output feature map. Equation (8) represents the calculation of the regression branch, and Conv_Reg represents the convolutional layer dedicated to the regression branch. Equation (9) represents the classification branch calculation, and Conv represents the general convolutional layer. Equation (10) represents the scaling of regression results, and Scale represents the scaling operation, which involves proportionally adjusting the regression branch results to adapt to different scale targets. Equation (11) represents feature concatenation; Concat represents the feature concatenation operation, concatenate along dimension dim=1. Equation (12) represents the multi-scale feature output during the training phase.

(6) $$x_{i}^{conv}=Conv\text{\_}GN(x_{i}),$$

(7) $$x_{i}^{share}=DEConv\text{\_}GN(DEConv\text{\_}GN(x_{i}^{conv})),$$

(8) $$Reg_{i}=Conv\text{\_}Reg(x_{i}^{share}),$$

(9) $$Cls_{i}=Conv(x_{i}^{share}),$$

(10) $$Reg_{i}^{scale}=Scale(Reg_{i}),$$

(11) $$x_{i}=Concat(Reg_{i}^{scale},Cls_{i},dim=1),$$

(12) $$output_{train}=[x_{i},x_{i+1},x_{i+2}],$$

**Figure 5:**
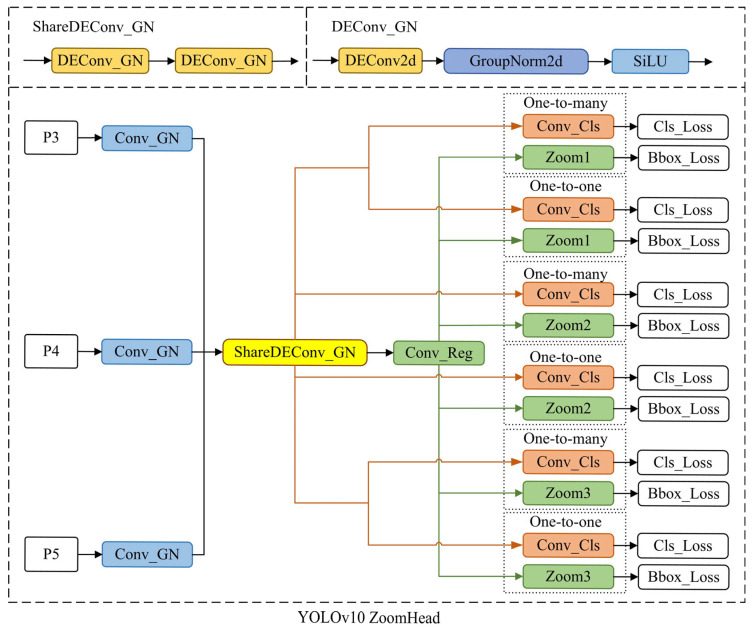
Schematic diagram of YOLOV10 ZoomHead structure.

The structure in Figure 4b applies to the YOLO11 model. If the ZoomHead structure needs to be applied to YOLOv10, the method proposed in this paper needs only to be retained. The original regression and classification branches can be adjusted accordingly to the one-to-one and one-to-many branches, as shown in Figure 5.

## 4. Experiments, Results, and Analysis

### 4.1. Experimental Setup

#### 4.1.1. Dataset

This article conducts model training and performance comparison on the proposed YOLO-ZoomHead structure based on two datasets and sequentially verifies and explores its detection and classification performance.

1. The dataset of rail surface micro-cracks from the Internet is used as the training and verification sample set, which contains more than 2000 original images with a size of (1200×1400×3). We manually removed images with poor quality (such as high or low contrast, clarity, etc.) and images that do not meet the characteristics of small cracks (such as large crack width) and retained a total of 1000 images. Subsequently, the Labelme library in the Python 3.9.21 environment was used to perform target annotation operations on the selected 1000 data images.

**Figure 6:**
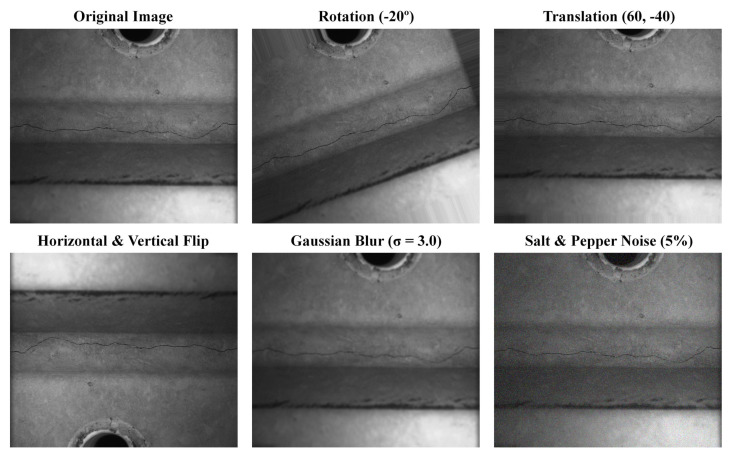
Partial small crack image samples.

In order to fully enhance the diversity of defect samples in the dataset and indirectly improve the efficiency and performance of subsequent model training, it is necessary to perform enhancement operations on the original dataset. This article selects ImageAug, a third-party image processing tool in a Python environment, to perform data augmentation on the 1000 images that have undergone object annotation operations mentioned above. ImageAug 0.4.0 can perform various single or combined lossless enhancement operations on images such as rotation, translation, cropping, color space adjustment, distortion, blurring, and filtering. At the same time, it can synchronously transform the bounding boxes in the image, that is, enhance the previous annotation information together to ensure that the enhanced defect sample images have accurate standard information. It has wide applications in the enhancement process of object detection task datasets. When performing data augmentation operations in this article, each original image was subjected to one enhancement operation, and the specific enhancement method was randomly selected by the algorithm. Finally, 1000 enhanced images were obtained and included in the dataset along with the previous 1000 original images. This study obtained a total of 2000 sample datasets of micro-crack images, some of which are shown in Figure 6.

To increase the proportion of samples used for training as much as possible, the 2000 pieces of data were randomly divided according to a ratio of 9:0.5:0.5 between the training set, validation set, and test set. The final result showed that the training set contained 1800 images, and the validation set and test set contained 100 images each.

**Figure 7:**
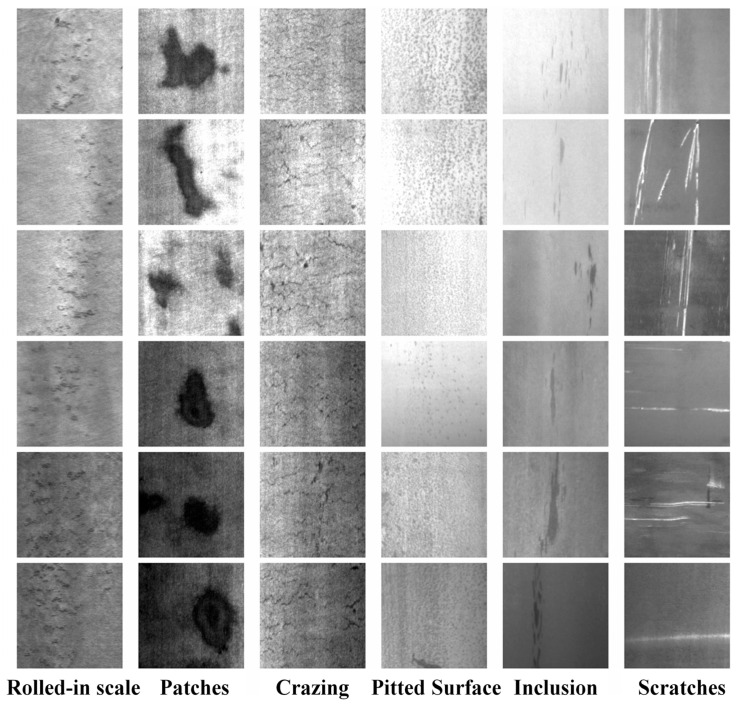
Partial defect images of strip steel.

2. We chose the NEU surface defect database, which Professor Song Kechen led from Northeastern University to organize defect image data on the surface of hot-rolled steel strips [32]. Six typical surface defects of hot-rolled steel strips were collected: rolled-in scale, patches, crazing, pitted surface, inclusions, and scratches. There were 300 defect images for each category, totaling 1800 defect images, each image with a size of (200 × 200 × 1). Partial defect images of strip steel in this dataset are shown in Figure 7.

#### 4.1.2. Experiment Settings

A stable, reliable, and high-performance computing hardware platform is the foundation of model training. In this paper, the DELL graphics workstation (DELL Precision T7920) is selected as the training carrier for all network models. The specific hardware parameters and model training environment information are shown in Table 2.

#### 4.1.3. Evaluation Metrics

To accurately evaluate the effectiveness of the proposed improvement plan for the model in this article, it is necessary to establish evaluation indicators for the model’s performance comparison experiments in advance. This article uses precision (P), recall (R), mean average precision (mAP), parameter count (Params), floating-point operation count (GFLOPs), and detection speed (frames per second, FPS) as evaluation indicators for the metal fine crack defect detection model. P is used to evaluate the degree of false detection, R is used to evaluate the situation of missed detection, the average accuracy at an IoU threshold of 0.5 mAP@50 and when the IoU threshold varies between 0.5 and 0.95 mAP@50-95 is used to evaluate model performance, Params and GFLOPs are used to assess model size and computational resource requirements, while FPS is used to evaluate model response and inference speed.

The confusion matrix is shown in Table 3, where *TP* represents true positives, which is the number of samples correctly predicted as positive by the model, *FP* represents false positives, which is the number of samples incorrectly predicted as positive by the model, *FN* represents false negatives, which is the number of samples incorrectly predicted as negative by the model, and *TN* represents true negatives, which is the number of samples correctly predicted as negative by the model. The calculation formulas for *P* and *R* are, respectively, as follows:

(13) $$P=\frac{TP}{TP+FP},$$

(14) $$R=\frac{TP}{TP+FN}$$

In the formula, *TP* is true positive, *FP* is false positive, and *FN* is false negative.

*AP* is the area under the *P*-*R* curve, representing the average accuracy, while mAP represents the mean *AP* value of all categories. The closer its value is to 1, the better the model’s detection performance for targets. The calculation formula is

(15) $$AP=\int_{0}^{1}P\left(R\right)DR,$$

(16) $$R=\frac{TP}{TP+FN}$$

**Table 3:** Confusion matrix.

Confusion Matrix		Ground Truth	
		True	False
**Predicted Value**	Positive	TP	FP
	Negative	FN	TN

#### 4.1.4. Baseline Models

All model comparison experiments in this section selected the YOLOv10 series and YOLO11 series models as baseline models. The ZoomHead structure proposed in Section 3.3 was deployed in the baseline model to replace the original head detection head structure as a whole. Subsequently, a series of performance comparison experiments was conducted to verify the effectiveness and reliability of the ZoomHead structure.

### 4.2. Experimental Analysis and Verification

To verify the effectiveness of the proposed ZoomHead detection head structure at improving the detection of metal micro-crack defects, a performance comparison experiment was conducted between the YOLO-ZoomHead object detection model and YOLOv10 and YOLO11 deep learning object detection models of different scales. Model performance comparison experiments were conducted using the rail surface crack dataset and the NEU surface defect database. And we evaluated the performance of different object detection models based on indicators such as P, R, mAP@50, mAP@50-95, Params, GFLOPs, and FPS. The arrows in the experimental results table are used to indicate the improvement or reduction of evaluation indicators. For example, if the P of YOLOv10n increases from 0.660 to 0.674, it can be expressed as (↑0.014), and if Params decreases from 2.265M to 1.943M, it can be expressed as (↓0.322M). This expression also applies to Table 4, Table 5, Table 6, Table 7, Table 8, Table 9, Table 10 and Table 11.

#### 4.2.1. Comparative Experiment on Model Performance Based on Rail Surface Crack Dataset

To verify the effectiveness of YOLO-ZoomHead in improving detection performance, this section conducted performance comparison experiments based on the rail surface crack dataset. The experimental results are shown in Table 4.

From Table 4, it can be seen that regardless of the size of the model, compared with the original baseline model, the YOLO-ZoomHead object detection model has improved to a certain extent in terms of P, R, and mAP values after adding the ZoomHead structure, indicating that the model can more accurately identify target defects, effectively reduce missed detection rates, and improve model detection performance. ZoomHead shows significant optimization effects on evaluation metrics such as Params and GFLOPs. Taking the YOLOv10 model as an example, the key indicators of some models show a significant decrease. After introducing the ZoomHead structure into YOLOv10n, its Params decreased by 0.322M, a decrease of 14.2%. After adding the ZoomHead structure to YOLOv10m, its GFLOPs decreased by 8.4, a decrease of 14.4%. These analyses indicate that ZoomHead can reduce redundant parameters and computational complexity, making the model more lightweight. At the same time, the FPS also has been improved to a certain extent. After adding the ZoomHead structure to YOLOv10m, its FPS increased by 6.4, an increase of 7.3%, indicating that the model has significant advantages in response and inference speed. Similarly, the YOLO11 model also exhibits similar patterns. Based on the above analysis, the performance comparison experiment on the rail surface crack dataset proves that the ZoomHead structure can fully balance the requirements between detection accuracy and speed and effectively improve the comprehensive detection performance of the model.

Furthermore, by comparing and analyzing the inference results of models of different scales, it was found that the comprehensive improvement effect of ZoomHead will show an overall increasing trend with the increase in model scale. This may be because, as the model scale increases, the feature maps from the neck structure contain more effective micro-crack feature information, making the introduction of the DEConv module and GroupNorm method more effective at improving detection performance. At the same time, the streamlined structure of ZoomHead effectively alleviates the sensitivity of redundant structures due to the increase in model computation, resulting in a significant improvement in model inference speed and better real-time performance.

**Figure 8:**
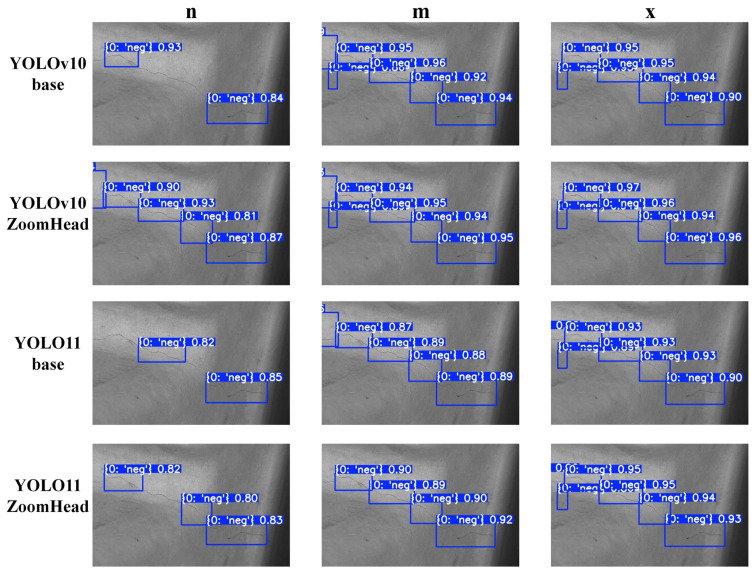
Visual comparison chart of model inference results.

To more intuitively demonstrate the improvement effect of the YOLO-ZoomHead model proposed in this paper on the detection performance of the baseline model, all trained models in Table 4 were used for inference analysis on the test set data samples. Table 4 demonstrates that the ZoomHead structure enhances the detection performance of YOLOv10/11 models of different scales; in the selection of model scale during the visualization stage, considering the clarity and representativeness of the visualization effect, we focus on selecting three typical scales of models: “n (lightweight version),” “m (medium version),” and “x (complete version)” for visualization display. This avoids visual confusion in the visualization results and comprehensively presents the effective improvement effect of the ZoomHead structure by covering different scale architectures. Visualize and compare the inference results of the models under the three scales mentioned above and obtain Figure 8. Figure 8 shows that the small-scale YOLOv10/11n-ZoomHead has a lower missed detection rate and higher crack target confidence compared with YOLOv10/11n base, while the medium and large-scale YOLOv10/11m/x-ZoomHead further improves crack target confidence compared with YOLOv10/11m/x-base. It can be seen that the addition of the ZoomHead structure effectively enhances the model’s detection performance.

**Table 4:** Experimental results of performance comparison of different object detection models.

Model	P	R	mAP@50	mAP@50-95	Params	GFLOPs	FPS
YOLOv10n	0.660	0.589	0.614	0.316	2.265M	6.5	719.7
YOLOv10n-ZoomHead	0.674 (↑0.014)	0.593 (↑0.004)	0.620 (↑0.006)	0.325 (↑0.009)	1.943M (↓0.322M)	6.2 (↓0.3)	726.4 (↑6.7)
YOLOv10s	0.752	0.648	0.721	0.412	7.218M	21.4	325.0
YOLOv10s-ZoomHead	0.788 (↑0.036)	0.666 (↑0.018)	0.734 (↑0.013)	0.420 (↑0.008)	6.821M (↓0.397M)	20.8 (↓0.6)	337.1 (↑12.1)
YOLOv10m	0.776	0.716	0.746	0.467	15.314M	58.9	151.8
YOLOv10m-ZoomHead	0.786 (↑0.010)	0.729 (↑0.013)	0.757 (↑0.011)	0.476 (↑0.009)	14.776M (↓0.538M)	50.4 (↓8.5)	154.9 (↑3.1)
YOLOv10b	0.803	0.732	0.782	0.521	19.005M	91.6	111.5
YOLOv10b-ZoomHead	0.829 (↑0.026)	0.751 (↑0.019)	0.797 (↑0.015)	0.527 (↑0.006)	18.624M (↓0.381M)	87.8 (↓3.8)	117.4 (↑5.9)
YOLOv10l	0.751	0.742	0.725	0.470	24.310M	120.0	88.2
YOLOv10l-ZoomHead	0.775 (↑0.024)	0.767 (↑0.025)	0.764 (↑0.039)	0.499 (↑0.029)	23.829M (↓0.481M)	115.2 (↓4.8)	94.6 (↑6.4)
YOLOv10x	0.768	0.713	0.755	0.499	29.397M	160.0	58.2
YOLOv10x-ZoomHead	0.778 (↑0.010)	0.741 (↑0.028)	0.773 (↑0.018)	0.526 (↑0.027)	29.088M (↓0.309M)	153.6 (↓6.4)	61.1 (↑2.9)
YOLO11n	0.656	0.546	0.561	0.281	2.582M	6.3	733.3
YOLO11n-ZoomHead	0.663 (↑0.007)	0.552 (↑0.006)	0.569 (↑0.008)	0.288 (↑0.007)	2.260M (↓0.322M)	6.0 (↓0.3)	744.1 (↑10.8)
YOLO11s	0.733	0.626	0.670	0.369	9.413M	21.3	309.8
YOLO11s-ZoomHead	0.743 (↑0.010)	0.665 (↑0.039)	0.681 (↑0.011)	0.386 (↑0.017)	9.015M (↓0.398M)	20.7 (↓0.6)	320.5 (↑10.7)
YOLO11m	0.764	0.704	0.733	0.471	20.031M	67.6	120.4
YOLO11m-ZoomHead	0.795 (↑0.031)	0.726 (↑0.022)	0.757 (↑0.024)	0.484 (↑0.013)	19.750M (↓0.281M)	64.8 (↓2.8)	125.4 (↑5.0)
YOLO11l	0.801	0.724	0.751	0.505	25.281M	80.6	93.5
YOLO11l-ZoomHead	0.826 (↑0.025)	0.734 (↑0.010)	0.763 (↑0.012)	0.519 (↑0.014)	25.099M (↓0.182M)	75.7 (↓4.9)	99.0 (↑5.5)
YOLO11x	0.811	0.734	0.751	0.522	56.828M	194.4	47.9
YOLO11x-ZoomHead	0.819 (↑0.008)	0.746 (↑0.012)	0.783 (↑0.032)	0.554 (↑0.032)	56.509M (↓0.319M)	189.4 (↓5.0)	49.5 (↑1.6)

#### 4.2.2. Performance Comparison Experiment of Models Based on NEU Surface Defect Database

To further validate the generalization performance of the YOLO-ZoomHead algorithm, performance comparison experiments were conducted on different models based on the NEU surface defect database. The experimental results are shown in Table 5. Due to the constant and unchanged comparison between the model architecture and the testing hardware environment in this experiment, Params and GFLOPs are inherent properties of the model, and FPS is used as an inference efficiency indicator under a stable hardware environment. The results of these three indicators are completely consistent with the experimental results in Table 4. Therefore, to avoid redundancy in the presentation of experimental results, Table 5 focuses on analyzing indicators strongly correlated with the dataset, including P, R, mAP@50, and mAP@50-95. Similarly, when conducting experiments based on the NEU surface defect database, Table 9 and Table 11 follow the same principle and present only data-driven indicators.

**Figure 9:**
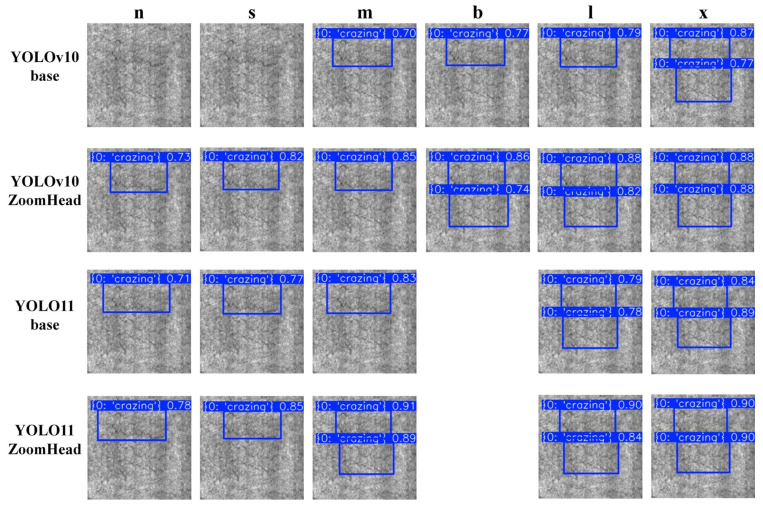
Visualization of model reasoning results for Crazing categories.

The experimental results in Table 5 show that compared with the baseline model, the proposed YOLO-ZoomHead model has shown a basic improvement effect in various detection performance indicators, which is consistent with the experimental results in Table 4. However, it should be noted that for some models, such as YOLOv10m, YOLOv10l, etc., there was a slight decrease in mAP values, while YOLOv10b, YOLOv10l, YOLO11s, YOLO11x, etc. showed a slight decrease in P or R values. This is because the NEU surface defect database is a six-class dataset, and the mAP values in Table 5 are the average of mAP values for different categories. However, by examining the mAP@50-95 values for each category of the YOLOv10m model in more detail (as shown in Table 6), it can be found that the addition of the ZoomHead structure increases the mAP values for Patches, Inclusion, Scratches, and other categories, while the mAP values for the Crafting, Pitted Surface, and Rolled in Scale categories slightly decrease. Based on Figure 9, it can be observed that the addition of the ZoomHead structure improves the detection performance of the model for patchy and strip-shaped targets that are close to crack morphology while weakening the detection performance in scenarios where the difference between foreground targets and background textures is small. The mAP@50-95 values for each category of the YOLOv10l model (as shown in Table 7) exhibited the same trend. For models such as YOLOv10b, YOLOv10l, YOLO11s, and YOLO11x, there was a slight decrease in P or R values, which was also observed under the categories of Crazing, Pitted Surface, and Rolled in Scale targets. Considering all factors, the addition of the ZoomHead structure did not result in an overall decrease in the detection performance of a particular model. Therefore, the ZoomHead structure is still effective at improving the detection performance of the model.

To further validate the overall effectiveness of the ZoomHead structure in the NEU surface defect database, a visual comparison was made between the training and inference results corresponding to Table 5, resulting in Figure 9. Among the six categories, the Crazing category, with the highest detection difficulty, was selected for visualization display. It can be seen that the baseline models of YOLOv10n and YOLOv10s have missed the detection of Crazing class targets. However, with the addition of the ZoomHead structure, the missed detection phenomenon has been alleviated. From Figure 9, regardless of the size of YOLOv10/11, adding the ZoomHead structure can improve its comprehensive detection performance for Crazing categories. Although the YOLOv10m/l model showed a slight decrease in mAP values, it still demonstrated visible performance improvement in the inference results of the test set, thereby proving the effectiveness of the structure.

**Table 5:** Experimental results of the performance comparison of different object detection models based on the NEU surface defect database.

Model	P	R	mAP@50	mAP@50-95
YOLOv10n	0.742	0.693	0.735	0.393
YOLOv10n-ZoomHead	0.787 (↑0.045)	0.702 (↑0.009)	0.767 (↑0.032)	0.412 (↑0.019)
YOLOv10s	0.739	0.689	0.738	0.403
YOLOv10s-ZoomHead	0.798 (↑0.059)	0.707 (↑0.018)	0.762 (↑0.024)	0.419 (↑0.016)
YOLOv10m	0.702	0.693	0.727	0.407
YOLOv10m-ZoomHead	0.727 (↑0.025)	0.711 (↑0.018)	0.744 (↑0.017)	0.404 (↓0.003)
YOLOv10b	0.762	0.705	0.744	0.400
YOLOv10b-ZoomHead	0.742 (↓0.020)	0.707 (↑0.002)	0.752 (↑0.008)	0.418 (↑0.018)
YOLOv10l	0.762	0.660	0.745	0.399
YOLOv10l-ZoomHead	0.753 (↓0.009)	0.664 (↑0.004)	0.737 (↓0.008)	0.407 (↑0.008)
YOLOv10x	0.711	0.684	0.744	0.411
YOLOv10x-ZoomHead	0.779 (↑0.068)	0.684	0.754 (↑0.010)	0.417 (↑0.006)
YOLO11n	0.695	0.744	0.748	0.416
YOLO11n-ZoomHead	0.734 (↑0.039)	0.762 (↑0.018)	0.762 (↑0.014)	0.422 (↑0.006)
YOLO11s	0.789	0.693	0.755	0.428
YOLO11s-ZoomHead	0.811 (↑0.022)	0.691 (↓0.002)	0.759 (↑0.004)	0.444 (↑0.016)
YOLO11m	0.742	0.710	0.757	0.416
YOLO11m-ZoomHead	0.769 (↑0.027)	0.740 (↑0.030)	0.776 (↑0.019)	0.423 (↑0.007)
YOLO11l	0.727	0.731	0.765	0.422
YOLO11l-ZoomHead	0.763 (↑0.036)	0.75 (↑0.019)	0.779 (↑0.014)	0.429 (↑0.007)
YOLO11x	0.775	0.687	0.759	0.409
YOLO11x-ZoomHead	0.770 (↓0.005)	0.688 (↑0.001)	0.770 (↑0.011)	0.419 (↑0.010)

**Table 6:** ZoomHead structure before and after adding YOLOv10m model for each category mAP@50-95 numerical value.

Model	All	Crazing	Patches	Inclusion	Pitted Surface	Rolled-in Scale	Scratches
YOLOv10m-base	0.407	0.157	0.53	0.392	0.565	0.235	0.563
YOLOv10m-ZoomHead	0.404	0.152	0.541	0.396	0.534	0.223	0.575

**Table 7:** ZoomHead structure before and after adding YOLOv10l model for each category mAP@50-95 numerical value.

Model	All	Crazing	Patches	Inclusion	Pitted Surface	Rolled-in Scale	Scratches
YOLOv10m-base	0.399	0.171	0.486	0.403	0.502	0.238	0.594
YOLOv10m-ZoomHead	0.407	0.169	0.517	0.426	0.490	0.236	0.601

### 4.3. Ablation Studies

Ablation experiments were conducted to validate further the effectiveness of the YOLO-ZoomHead algorithm proposed in this paper. The specific steps are to use GroupNorm, DEConv, and Zoom as independent variable modules and sequentially add the above modules based on the baseline models YOLOv10 and YOLO11. The impact of each module on detection performance is verified by comparing and analyzing the improvement effect of the experiment with indicators such as mAP value, Params, GFLOPs, and FPS. When conducting ablation experiments, we used only the “x” scale model because it has the maximum model depth and width at the “x” scale, which can contain more detailed information and thus more easily highlight the contribution differences of each optimization module.

#### 4.3.1. Ablation Experiment Based on Rail Surface Crack Dataset

Ablation experiments were conducted based on the rail surface cracks dataset, and the ablation experiments’ detection results are shown in Table 8.

By analyzing the data in Table 8, it can be concluded that the three sub-modules in the YOLO-ZoomHead structure proposed in this paper can each provide comprehensive performance improvement effects for the baseline model without interfering with each other, and the improvement effects can be stacked. Specifically, distribution module addition experiments were conducted using YOLOv10x and YOLO11x as baseline models. Firstly, the GN module was added to the YOLOv10x model, so that mAP@50 increased to 0.765 from 0.755 and mAP@50 from 0.499 to 0.511; there was almost no change in other indicators, indicating that the GN module has a significant improvement in model accuracy and almost does not increase the computational burden. Next, the addition of the DEConv module resulted in a slight increase in parameter and computational complexity as well as a slight decrease in inference speed. However, the mAP value showed a significant improvement, indicating that the addition of the DEConv module can significantly enhance the ability to detect small targets and capture details, while the increase in computational complexity is relatively small and controllable. The addition of the Zoom scale factor ensures model accuracy while reducing parameter count by 0.309M, computational complexity by 6.4, and FPS by 2.9, achieving dual optimization of model lightweight and inference efficiency. However, it should be noted that by adding the Zoom scale factor on top of GN and DEConv, the FPS of the model has been improved, but the mAP value has slightly decreased. The reason for this is that the Zoom scale factor uses the idea of proportional amplification to equate the output of one convolution kernel to three, and the equivalent process introduces some additional errors, ultimately resulting in a slight decrease in detection performance. However, considering that the addition of the Zoom scale factor effectively reduces computational redundancy, reduces the number of parameters and calculations in the model, and improves FPS, it can still be considered that the ZoomHead structure has sufficient effectiveness. Similarly, the YOLO11x model exhibited similar patterns in experiments. In summary, compared with the baseline model, the YOLO-ZoomHead model proposed in this paper can achieve a good balance between detection accuracy and efficiency in metal micro-crack defect detection and has a lower number of parameters and computational complexity, making it lighter for equipment deployment.

**Table 8:** YOLO-ZoomHead ablation experiments with different modules.

Model	GN	DEConv	Zoom	mAP@50	mAP@50-95	Params	GFLOPs	FPS
YOLOv10x				0.755	0.499	29.397M	160.0	58.2
	✓			0.765 (↑0.010)	0.511 (↑0.012)	29.405M (↑0.007M)	160.8 (↑0.8)	58.0 (↓0.2)
	✓	✓		0.775 (↑0.020)	0.527 (↑0.028)	29.422M (↑0.025M)	162.1(↑2.1)	56.8 (↓1.4)
	✓	✓	✓	0.773 (↑0.018)	0.526 (↑0.027)	29.088M (↓0.309M)	153.6 (↓6.4)	61.1 (↑2.9)
YOLO11x				0.751	0.522	56.828M	194.4	47.9
	✓			0.766 (↑0.015)	0.535 (↑0.013)	56.836M (↑0.008M)	194.9 (↑0.5)	47.2 (↓0.7)
	✓	✓		0.784 (↑0.033)	0.554 (↑0.032)	57.101M (↑0.273M)	196.5 (↑2.1)	45.5 (↓2.4)
	✓	✓	✓	0.783 (↑0.032)	0.554 (↑0.032)	56.509M (↓0.319M)	189.4 (↓5.0)	49.5 (↑1.6)

#### 4.3.2. Ablation Experiment Based on NEU Surface Defect Database

Ablation experiments were conducted based on the NEU surface defect database, and the detection results of the ablation experiments are shown in Table 9.

The data analysis in Table 9 shows that with YOLOv10x and YOLO11x as baseline models, three independent variable modules, GN, DEConv, and Zoom, were introduced in sequence. As the modules were gradually added, evaluation indicators such as mAP@50 and mAP@50-95 showed a continuous growth trend, indicating that the detection performance of the model has been continuously improved. Taking YOLOv10x as an example for analysis, after adding GN alone, its mAP@50 value increased from 0.744 to 0.748. Continuing to add DEConv, the mAP@50 value increased from 0.748 to 0.751, and then adding Zoom, the mAP@50 value increased from 0.751 to 0.754. Compared with the baseline model, the detection performance of the model has been significantly improved. YOLO11x exhibits similar patterns. Combining the ablation experimental results on the two datasets of Table 8 and Table 9 further proves the rationality of the YOLO-ZoomHead proposed in this paper.

**Table 9:** YOLO-ZoomHead ablation experiments with different modules based on NEU surface defect database.

Model	GN	DEConv	Zoom	mAP@50	mAP@50-95
YOLOv10x				0.744	0.411
	✓			0.748 (↑0.004)	0.412 (↑0.001)
	✓	✓		0.751 (↑0.007)	0.416 (↑0.005)
	✓	✓	✓	0.754 (↑0.010)	0.417 (↑0.006)
YOLO11x				0.759	0.409
	✓			0.766 (↑0.007)	0.411 (↑0.002)
	✓	✓		0.769 (↑0.010)	0.418 (↑0.009)
	✓	✓	✓	0.770 (↑0.011)	0.419 (↑0.010)

### 4.4. Comparative Experiment with SOTA Model

A comparative experiment with SOTA models was designed to verify the further advancement of YOLO ZoomHead. We selected SOTA models that optimize head structures and have been published at top conferences (e.g., CVPR, ICCV) in recent years, such as DyHead [33], EfficientHead [34], SEAMHead [35], and LQEHead [36]. Among them, DyHead is a dynamic routing detection head that adaptively allocates computing resources for different tasks through an attention mechanism, supporting the dynamic aggregation of cross-scale features. EfficientHead is a lightweight decoupled detection head that uses depthwise separable convolution and channel pruning strategies to reduce computational complexity while maintaining accuracy. SEAMHead is a detection head based on spatial channel dual attention, which enhances effective features to suppress noise and improve the detection performance of complex scenes. LQEHead is a low-quality feature enhancement detection head that integrates a feature repair network and a quality evaluation module to optimize small and fuzzy object detection. Then, they are deployed sequentially in the head structure of the YOLO11n model or perform overall replacement, using the same hyperparameters to train and reason for each model.

#### 4.4.1. Comparative Experiment Based on Rail Surface Crack Dataset

We conducted SOTA comparative experiments based on the steel rail surface crack dataset, and the results are shown in Table 10.

**Table 10:** Comparative experiments.

Model	mAP@50	mAP@50-95	Params	GFLOPs	FPS
YOLO11n-base	0.561	0.281	2.582M	6.3	733.3
YOLO11n-DyHead	0.570 (↑0.009)	0.289 (↑0.008)	3.099M (↑0.517M)	7.4 (↑1.1)	307.3 (↓426.0)
YOLO11n-EfficientHead	0.555 (↓0.006)	0.272 (↓0.009)	2.312M (↓0.270M)	5.1 (↓1.2)	710.9 (↓22.4)
YOLO11n-SEAMHead	0.565 (↑0.004)	0.285 (↑0.004)	2.491M (↓0.091M)	5.8 (↓0.5)	682.3 (↓51.0)
YOLO11n-LQEHead	0.558 (↓0.003)	0.275 (↓0.006)	2.587M (↑0.005M)	6.3	624.9 (↓108.4)
YOLO11n-ZoomHead	0.569 (↑0.008)	0.288 (↑0.007)	2.260M (↓0.322M)	6.0 (↓0.3)	744.1 (↑10.8)

Table 10 shows that the YOLO-ZoomHead model proposed in this paper exhibits better comprehensive detection performance than other SOTA models. Specifically, regarding mAP@50 and mAP@50-95, YOLO-ZoomHead ranks second only to YOLO11n-DyHead (with only a 0.1% difference). However, while DyHead ensures high accuracy, it has a larger parameter count and higher computational complexity, indicating significant computational costs. The FPS dropped significantly from 733.3 to 307.3, further confirming that there is still room for improvement in the real-time performance of the DyHead model. Regarding Params and FPS, YOLO-ZoomHead is significantly better than other algorithms, with a parameter count of 2.260M, 0.322M less than the benchmark model. The computational cost is significantly reduced, the model is lighter, and the inference speed is slightly improved. Regarding GFLOPs metrics, the EfficientHead and SEAMHead algorithms have lower computational complexity. However, compared with the ZoomHead algorithm proposed in this paper, their mAP values have decreased, resulting in poor overall detection performance of the models. In conclusion, the YOLO-ZoomHead algorithm proposed in this paper not only has significant advantages in detection speed but also realizes the balance between detection accuracy and model parameters, dramatically improves the computing speed, and meets the lightweight deployment requirements of object detection in embedded edge computing devices.

**Figure 10:**
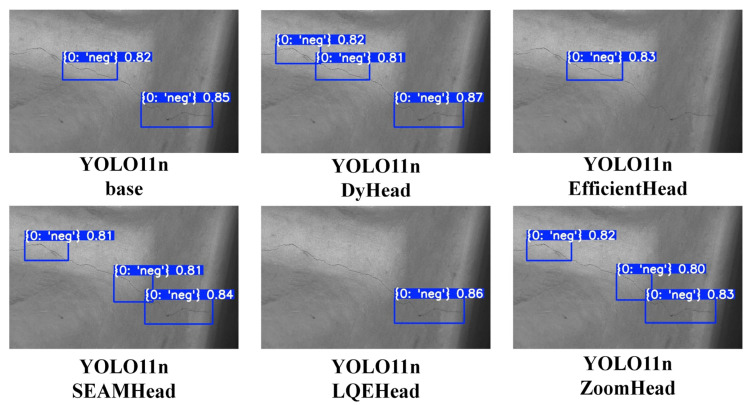
Visual comparison chart of SOTA model inference results.

To more intuitively demonstrate the performance differences between the YOLO-ZoomHead model and SOTA model proposed in this article, all models in Table 10 were used to infer the test set image samples, and the results were visually compared, resulting in Figure 10. It can be seen that the EfficientHead and LQEHead models did not show significant improvement compared with the baseline model, while the DyHead and SEAMHead models improved the missed detection rate and target confidence compared with the baseline model. The ZoomHead model has crack detection performance that is basically equivalent to the Dyhead and SEAMHead models (corresponding to the mAP values in Table 10). Combining the FPS of Dyhead and SEAMHead models in Table 10, which are both lower than ZoomHead, it can be proved that the ZoomHead proposed in this paper has the best comprehensive crack detection performance.

#### 4.4.2. Comparative Experiment Based on NEU Surface Defect Database

We conducted SOTA comparative experiments based on the NEU surface defect database, and the results are shown in Table 11.

**Table 11:** Comparative experiments based on NEU surface defect database.

Model	mAP@50	mAP@50-95
YOLOv11n-base	0.748	0.416
YOLOv11n-DyHead	0.755 (↑0.011)	0.419 (↑0.003)
YOLOv11n-EfficientHead	0.747 (↓0.001)	0.416
YOLOv11n-SEAMHead	0.752 (↑0.004)	0.421 (↑0.005)
YOLOv11n-LQEHead	0.742 (↓0.006)	0.411 (↓0.005)
YOLOv11n-ZoomHead	0.772 (↑0.024)	0.422 (↑0.006)

By analyzing the data in Table 11, it can be intuitively seen that the proposed YOLO-ZoomHead model is significantly better than other models in terms of the indicators mAP@50 and mAP@50-95. Combined with the data in Table 10, it can be seen that it has the lowest number of parameters and the highest FPS, and the computational complexity has also been reduced to a certain extent. In summary, the comparative experiments on the NEU-DET dataset indicate that YOLO-ZoomHead achieves the optimal balance between detection accuracy, parameter quantity, and resource efficiency. Meanwhile, compared with other lightweight models such as EfficientHead, the proposed YOLO-ZoomHead significantly improves detection performance while maintaining low computational complexity and parameter count.

**Figure 11:**
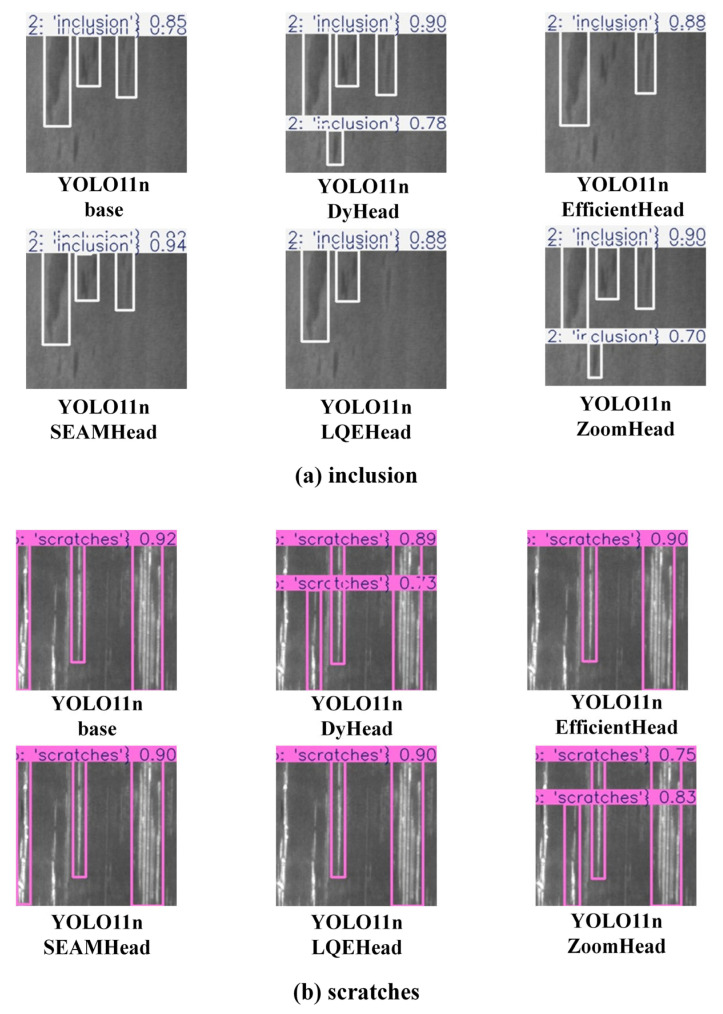
Visual comparison chart of model inference results for inclusions and scratches.

To more intuitively demonstrate the performance differences between the YOLO-ZoomHead model proposed in this article and the SOTA model, all models in Table 11 were used to infer the test set image samples, and the results were visualized for comparison. We focused on selecting the inclusion and scratch categories close to the crack morphology and obtained Figure 11. It can be seen that in the NEU surface defect dataset, the proposed model still exhibits almost the same trend as the rail crack dataset. That is, the detection accuracy of the YOLO11n-ZoomHead model is slightly inferior to the YOLO11n-DyHead model but significantly better than the others. Based on the data in Table 10, it can be concluded that the Dyhead model has higher computational and parameter complexity than ZoomHead, and its FPS is much lower than ZoomHead. Based on the above analysis, it can be proven that the ZoomHead proposed in this paper has the best comprehensive crack detection performance.

## 5. Conclusions

To effectively solve the problems of difficulty in accurately identifying small cracks on the surface of metal, difficulty in deploying edge computing equipment due to the large number of parameters and calculations in existing algorithm models, difficulty in balancing model accuracy and speed, and difficulty in meeting the safety operation requirements of industrial equipment, this paper proposes an improved lightweight detection method, YOLO-ZoomHead, with high precision and high speed based on the existing YOLO architecture.

Firstly, this method stabilizes the feature distribution, accelerates training convergence, and enhances model generalization ability by replacing BatchNorm2d with GroupNorm2d. Additionally, it replaces the traditional convolution kernel with DEConv and modifies it to a shared convolution structure to reduce redundant structures. Furthermore, the Zoom scale factor is introduced to perform relatively free proportional scaling operations on the convolution kernels in the regression branch, effectively reducing the parameter and computational complexity and improving model response speed. Subsequently, the proposed algorithm was deployed in baseline models such as YOLOv10 and YOLO11, and a series of performance comparison experiments were conducted on the rail surface crack dataset and NEU surface defect database. The results showed that adding ZoomHead effectively improved the model accuracy compared with the baseline model, regardless of the model size. It can achieve varying degrees of improvement in precision, recall, mAP value, and other indicators while effectively reducing the model’s parameters and computational complexity. The maximum reduction in the number of parameters for the detection head model can be 0.538M, and the maximum reduction in GFLOPs can be 8.4, significantly reducing the computational cost of the model. In addition, the FPS is significantly improved, reaching 12.1, improving the model response and inference speed, fully balancing the requirements between detection accuracy and speed, and verifying the effectiveness of the method proposed in this paper. Finally, in the comparative experiment of the SOTA model, the addition of ZoomHead resulted in the model having the smallest number of parameters and the highest FPS while maintaining the same mAP value as the SOTA model. This proves that the ZoomHead structure proposed in this paper can improve the comprehensive detection performance of the baseline model.

In summary, the YOLO-ZoomHead model proposed in this research achieves high-precision and high-speed detection of micro-cracks on metal surfaces while significantly reducing model parameters and computational complexity, effectively balancing the requirements between detection accuracy and speed. Its lightweight architecture substantially enhances hardware compatibility for practical deployment, enabling implementation on low-cost, low-power embedded edge devices while demonstrating excellent adaptability to complex industrial scenarios. This research can provide certain algorithmic support for the rational deployment of sensor hardware and feasible guarantees for real-time processing of multimodal data fusion. Future studies will further explore the synergistic optimization between object detection algorithms and multimodal sensors to advance the intelligent upgrading of industrial inspection systems.

## Figures and Tables

**Table 1:** Proportion of head structure.

Model	Head	All
YOLOv5n	3.64 (47.3%)	7.7
YOLOv8n	3.64 (41.8%)	8.7
YOLOv10n	3.8 (56.7%)	6.7
YOLO11n	1.9 (29.2%)	6.5

**Table 2:** Experiment settings.

Category	Detail Information
CPU	Intel(R) Xeon(R) E5-2698v3
GPU	Tesla V100 16 GB × 2
RAM	64 GB
Operating System	Ubuntu 22.04
Operating Environment	Anaconda3, CUDA12.1, Python3.9
Deep Learning Framework	PyTorch 2.2.2

## Data Availability

The data presented in this study are available on request from the authors.
